# Ischemic colitis caused increased early and delayed mortality

**DOI:** 10.1186/s13017-018-0193-2

**Published:** 2018-07-11

**Authors:** Hayim Gilshtein, Kenan Hallon, Yoram Kluger

**Affiliations:** Department of General Surgery, Rambam Health Care Campus, Hayim Gilshtein, 8 Haalia Street, Haifa, Israel

**Keywords:** Ischemic colitis, Mortality, Colonoscopy, Computed tomography

## Abstract

**Background:**

Ischemic colitis remains a challenge for the surgeon, both in its diagnosis and treatment. Data from a single tertiary center, of patients diagnosed with ischemic colitis, was collected. An attempt was made to delineate the patients requiring surgical intervention.

**Methods:**

A retrospective study was undertaken in patients diagnosed with ischemic colitis admitted to Rambam Health Care Campus between 2011 and 2016. The primary outcome was defined as mortality. Secondary outcomes were defined as complications during conservative treatment and postoperative course.

**Results:**

Sixty-three patients were diagnosed with ischemic colitis during the study period. The mean age at presentation was 72.5 years, with a female predominance (62%). The overall mortality rate was 29% (18/63). Six patients (50%) of those operated died. An older age, comorbidities and higher lactate levels present risk factors for a worse outcome.

**Conclusions:**

Ischemic colitis continues to present a challenge in its management. A better understanding of the disease process is required. And one needs to adhere to sound surgical principles for a timely diagnosis and treatment, especially in older patients with worrisome clinical, laboratory, and imaging features.

## Background

Ischemic colitis (IC) refers to the inflammation of the colon secondary to vascular insufficiency and ischemia [1, 2]. IC is the most common type of intestinal ischemia with an annual incidence of 15.6 to 17.7 per 100,000 [1]. Impaired perfusion of blood to the bowel from a variety of causes is the underlying pathophysiology. The diverse causes, variable clinical presentations, and severity make the diagnosis and management of ischemic colitis a challenge. The presence of diarrhea, abdominal pain, and mild lower gastrointestinal bleeding should prompt consideration of ischemic colitis as a causative etiology. IC is most prevalent in the elderly, in patients with multiple comorbid conditions, and in women.

The etiology of IC is multifactorial, and the clinical presentation varies upon the severity of deprivation of the intestinal blood flow and the development of frank ischemia and necrosis. Occlusive and non-occlusive diseases are the major mechanisms [2]. The diagnostic workup is not outlined clearly in the literature with the clinical presentation being the cornerstone of diagnosis. The main diagnostic modalities used are computed tomography (CT) and colonoscopy.

Ischemic colitis may eventually result in bowel perforation, peritonitis, persistent bleeding, protein-losing colopathy, and symptomatic intestinal strictures [1]. Due to the nature of the affected patients and the disease process itself, the mortality rate is high [3–6]. Nonetheless, the majority of cases are treated conservatively with surgical resection of the affected segment performed in only nearly 20% of patients presenting to the hospital with IC [1, 7–9].

In this article, we describe the characteristics of patients diagnosed with IC in a single tertiary center. We examined the methods of diagnosis and treatment with a special emphasis on patients who underwent surgery. We have tried to define which patients are more likely to undergo surgery in order to improve future management in this group.

## Methods

A retrospective study was undertaken in patients diagnosed with ischemic colitis admitted to Rambam Health Care Campus between 2011 and 2016. Using the computerized database, all patients with the diagnosis of ischemic colitis were included. Patients’ demographics, presenting symptoms, methods of diagnosis, and treatment were reviewed (Table 1). The primary outcome was defined as mortality. Secondary outcomes were defined as variables having an effect on surgical intervention, patient survival, patient comorbidities, and the indications for surgery. An IRB institutional approval was obtained.

**Table 1:** Demographic characteristics

Age (mean ± STD, range)	72.5 ± 15 [38–96]
Gender (female)	39 (62%)
Surgery (yes)	12 (19%)
Colonoscopy	16 (25%)
Hemoglobin	12.3 ± 2.3 [12]
Leukocytes	13.5 ± 7.0 [12]
Lactate	2.4 ± 1.7 [2]
Death	18 (29%)
Median follow-up (years)	2.27

### Data analysis

Descriptive statistics in terms of mean, standard deviation (STD), median, percentiles, and ranges were performed to all the study parameters. Normal distributions of the quantitative parameters were assessed by the Kolmogorov–Smirnov test.

Differences between groups were demonstrated by *t* test, Mann Whitney *U* test, and Fisher exact test. Survival analysis was performed using the Log-rank test. The Kaplan–Meier method of censored data estimation was also applied. Statistical analysis was performed using SPSS version 21.

## Results

We identified 63 patients with colonic ischemia over 6 years. The mean age at presentation was 72.5 years, with a female predominance (62%). All patients underwent CT with PO and IV contrast for diagnosis, and 16 patients (25%) underwent a colonoscopy as well. Overall, 50 of 63 (79%) patients were treated non-operatively with bowel rest and antibiotics. Twelve patients (24%) of those managed non-operatively died. Twelve patients (19%) were operated, of these, six patients (50%) died. The overall mortality rate was 29% (18/63). Higher leukocyte levels were found to be the only statistically significant variable for a patient undergoing surgical intervention (Table 2). A rise in lactate was identified in a patient who died (Table 3); other demographic and laboratory factors had not been significantly different.

**Table 2:** Surgical intervention

	Surgery, *n* = 12	Without surgery, *n* = 51	*p* value
Age (mean ± STD, range)	72.8 ± 11.8 [75.5]	72.4 ± 15.6 [71]	0.94
Gender (Female)	6 (50%)	33 (65%)	0.51
Colonoscopy	3 (25%)	13 (25%)	1.00
Hemoglobin	11.8 ± 2.5	12.5 ± 2.3	0.37
Leukocytes	17.1 ± 7.2	12.6 ± 6.8	0.03
Lactate	3.2 ± 2.4 [2]	2.2 ± 1.4 [1.7]	0.13
Death	6 (50%)	12 (24%)	0.085

**Table 3:** Comparison between patients who survived and those who died

	Death, *n* = 18	Alive, *n* = 45	*p* value
Age (mean ± STD, range)	84.2 ± 7.8	67.8 ± 14.4	< 0.0001
Gender (female)	8 (44%)	31 (69%)	0.09
Colonoscopy	2 (1%)	14 (31%)	0.12
Hemoglobin	11.2 ± 2.4	12.8 ± 2.2	0.019
Leukocytes	13.9 ± 6.3	13.3 ± 7.3	0.76
Lactate	3.3 ± 1.9 [2.85]	1.97 ± 1.4 [1.5]	0.003

The majority of the patients suffered from significant comorbidities (Table 4), such as chronic renal failure (13/63) and ischemic heart disease (20/63).

**Table 4:** Patient comorbidities

Comorbidities	Number of patients
Hypertension	38
Diabetes	13
Ischemic heart disease	20
COPD	5
Chronic renal failure	13
Atrial fibrillation	6

## Discussion

**Fig. 1:**
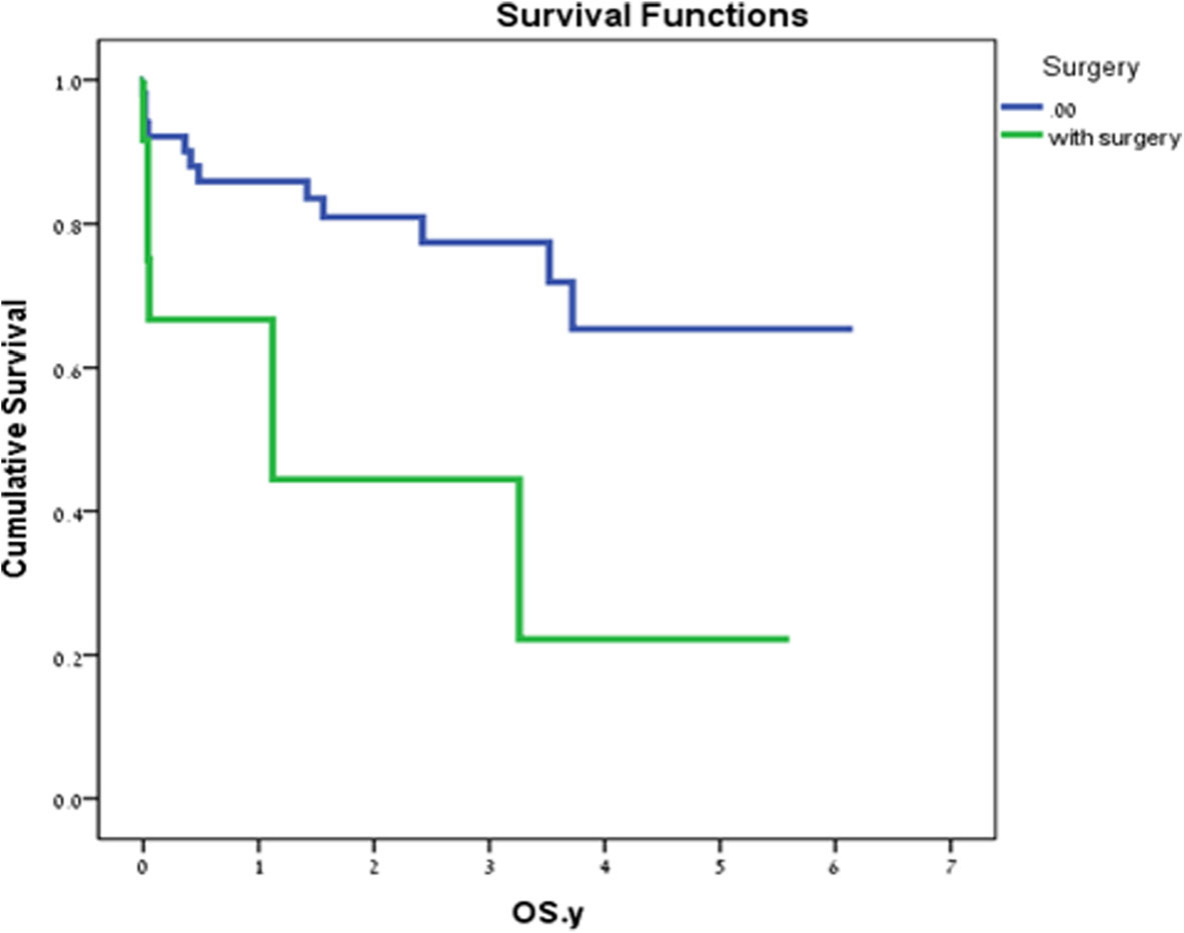
Kaplan–Meier curve comparing patients with and without surgery

The diagnosis of ischemic colitis is based on a high index of suspicion. Various diagnostic tests are implemented. While colonoscopy is considered the gold standard [10, 11], it has lower availability in the acute setting, especially in centers, such as ours where the endoscopies are performed by gastroenterologists. This explains the relatively low rate of diagnostic colonoscopies in our study. This makes the CT with an IV and oral contrast media the test of choice in our center and other reports [12–14]. In our study, the demographic characteristics of the patients were similar to previous reports. Also, the majority of the patients were treated non-operatively with about 20% operative rate, similar to previous reports. These patients were treated with NPO, monitored, and given IV antibiotics, third-generation cephalosporin, and metronidazole. Patients allergic to penicillin were treated with ciprofloxacin. The indications for surgery included the development of peritonitis, hemodynamic instability, and intractable disease (Table 5). The increased death rate of patients who underwent surgery reflects the severity of the disease in this group of patients (Table 4). The gap in mortality recorded in the acute phase is maintained and even increased in the extended follow-up period, beyond the acute presentation (Fig. 1). There are several possible explanations for this phenomenon. Primarily, it represents the disease severity upon presentation. Second, there is a close correlation with the patient’s comorbidities and age, as we can see with the statistically significant rise in mortality in the older age group (Table 3). But, in addition to these, we believe that there might be other detrimental factors in the disease process, etiology, and presentation not underlined thus far. The only laboratory factor found as a significant risk factor for mortality was increased lactate levels (Table 3). The disease presentation is variable without a clearly established treatment algorithm for the interesting entity of IC. With a pertinent anamnesis, a properly performed CT scan is adequate for establishing the diagnosis. A prompt surgical intervention is required for disease complications such as perforation and frank ischemia. However, one must bear in mind the older age group with comorbid diseases in which you might consider an earlier intervention even with more subtle signs such as higher lactate levels. An earlier surgery could improve the low patient survival rate in this complicated group. Additional studies are required to better determine which patients are prone to develop the much-dreaded complications requiring surgery. Preferably, these studies need to be multi-institutional and prospective in methodology with a higher recruited number of patients. We also believe that we need to try and look for a better understanding of the disease process, even at the microscopic level in order to understand the factors predisposing for a more serious outcome. Meanwhile, for the correct diagnosis and treatment, the surgeon needs to adhere to sound surgical principles and judgment with the aid of available diagnostic modalities. With the significant role of colonoscopy, we would like to stress its importance as a tool in the hands of the competent surgeon, requiring wider acceptance in countries worldwide, such as ours, where it remains in the sole position of the gastroenterologists.

**Table 5:** Indications for surgery

Indication	Number of patients
Peritonitis	6
Sepsis with hemodynamic compromise	4
Intractable disease	2

## Conclusions

Ischemic colitis causes increased mortality rates both in the immediate and late postoperative course. The disease process needs a better understanding in order to tailor the appropriate treatment, especially surgical intervention for those patients who require it.

## Abbreviations

CT: Computed tomography
IC: Ischemic colitis

## References

[1] Netz U, Galandiuk S (2017). The management of ischemic colitis. Current surgical therapy.

[2] Mahmoud NN, Bleier JIS, Aarons CB, Paulson EC, Shanmugan S, Fry RD (2017). Colon and Rectum. Sabiston textbook of surgery.

[3] Sun D, Wan C, Yang L, Liu M, Chen F (2016). The predictors of the severity of ischemic colitis: a systematic review of 2823 patients from 22 studies. Color Dis.

[4] Yadav S, Dave L (2015). A population-based study of incidence, risk factors, clinical spectrum, and outcomes of ischemic colitis. Clin Gastroenterol Hepatol.

[5] Díaz Nieto R, Varcada M, Ogunbiyi OA, Winslet MC (2011). Systematic review on the treatment of ischemic colitis. Color Dis.

[6] O’Neill S, Yalamarthi S (2012). Systematic review of the management of ischemic colitis. Color Dis.

[7] Brandt LJ, Feuerstadt P, Longstreth GF, Boley SJ, American College of Gastroenterology (2015). ACG clinical guideline: epidemiology, risk factors, patterns of presentation, diagnosis, and management of colon ischemia (CI). Am J Gastroenterol.

[8] Green BT, Tendler DA (2005). Ischemic colitis: a clinical review. South Med J.

[9] Castleberry AW, Turley RS, Hanna JM, Hopkins TJ, Barbas AS, Worni M (2013). A 10-year longitudinal analysis of surgical management for acute ischemic colitis. J Gastrointest Surg.

[10] Fitzgerald JF, Hernandez LO (2015). Ischemic colitis. Clin Colon Rectal Surg.

[11] Feuerstadt P, Brandt LJ (2010). Colon ischemia: recent insights and advances. Curr Gastroentrol Rep.

[12] Balthazar E, Yen B, Gordon R (1999). Ischemic colitis: CT evaluation of 54 cases. Radiology.

[13] Romano S, Romano L, Grassi R (2007). Multidetector row computed tomography findings from ischemia to infarction of the large bowel. Eur J Radiol.

[14] Menke J (2010). Diagnostic accuracy of multidetector CT in acute mesenteric ischemia: systematic review and meta-analysis. Radiology.

